# High-Frequency Transcutaneous Peripheral Nerve Stimulation Induces a Higher Increase of Heat Pain Threshold in the Cutaneous Area of the Stimulated Nerve When Confronted to the Neighbouring Areas

**DOI:** 10.1155/2013/464207

**Published:** 2013-08-06

**Authors:** M. Buonocore, N. Camuzzini, M. Cecini, E. Dalla Toffola

**Affiliations:** ^1^Salvatore Maugeri Foundation, Scientific Institute of Pavia, Unit of Clinical Neurophysiology & Neurodiagnostic Skin Biopsy, Via S. Maugeri 10, 27100 Pavia, Italy; ^2^Department of Rehabilitation Medicine, Ospedale S. Corona, Pietra Ligure, Savona, Italy; ^3^Physical Medicine and Rehabilitation Unit, Fondazione Policlinico San Matteo, Pavia, Italy

## Abstract

*Background*. TENS (transcutaneous electrical nerve stimulation) is probably the most diffused physical therapy used for antalgic purposes. Although it continues to be used by trial and error, correct targeting of paresthesias evoked by the electrical stimulation on the painful area is diffusely considered very important for pain relief. *Aim*. To investigate if TENS antalgic effect is higher in the cutaneous area of the stimulated nerve when confronted to neighbouring areas. *Methods*. 10 volunteers (4 males, 6 females) underwent three different sessions: in two, heat pain thresholds (HPTs) were measured on the dorsal hand skin before, during and after electrical stimulation (100 Hz, 0.1 msec) of superficial radial nerve; in the third session HPTs, were measured without any stimulation. *Results*. Radial nerve stimulation induced an increase of HPT significantly higher in its cutaneous territory when confronted to the neighbouring ulnar nerve territory, and antalgic effect persisted beyond the stimulation time. *Conclusions*. The location of TENS electrodes is crucial for obtaining the strongest pain relief, and peripheral nerve trunk stimulation is advised whenever possible. Moreover, the present study indicates that continuous stimulation could be unnecessary, suggesting a strategy for avoiding the well-known tolerance-like effect of prolonged TENS application.

## 1. Introduction 

Transcutaneous electrical nerve stimulation (TENS) is widely used all around the world for relieving a variety of painful conditions. Controlled clinical trials have clearly demonstrated that TENS has a specific antalgic effect [1–4], but the intrinsic mechanism remains largely unknown [5]. For this reason, on the clinical ground, TENS is largely used by trial and error, and the optimal setting of stimulation parameters is still a matter of debate.

A correct targeting of paresthesias evoked by the electrical stimulation on the painful area is commonly considered very important for pain relief. This seems to be confirmed by animal studies showing the higher antalgic effectiveness during the stimulation of peripheral nerve fibres afferent to the same spinal cord segment of the sensory fibers innervating the body part in pain [6]. The importance of a selective stimulation during TENS application for antalgic purposes has been rarely investigated in humans.

One of the best ways to better target the TENS effect is to induce a selective stimulation of a sensory nerve trunk, a method that permits to clearly identify the territory of evoked paresthesias, which exactly coincides with the cutaneous receptive field of the stimulated nerve. In this respect, it is important to underline that the term TENS embraces a variety of different techniques that use electrical stimulation of the skin for pain control [7]. The term HF-TPNS (high-frequency transcutaneous peripheral nerve stimulation) identifies a subtype of TENS where the trunk of a peripheral nerve is electrically stimulated at high frequency using surface electrodes [8].

The aim of the present study was to investigate, in a group of normal subjects, if the TENS antalgic effect is higher in the cutaneous area of the stimulated nerve when confronted to neighbouring areas.

## 2. Materials and Methods

### 2.1. Subjects

Unpaid human volunteers were recruited among the health care workers of our hospital. They were enrolled according to the following inclusion/exclusion criteria. Inclusion criteria: (a) at least 18 years of age; (b) right-handedness. Exclusion criteria: (a) history of peripheral neuropathy, trauma, surgery, and pain involving the arms; (b) use of current medications; (c) previous use of TENS; (d) pregnancy.

### 2.2. Heat Pain Threshold

The heat pain thresholds were studied using the Quantitative Sensory Testing (QST) according to the Marstock method [9–12]. Thermal stimuli were applied in a conditioned room (24–26°C) through a Peltier contact thermode with a surface of 12.5 cm^2^ (5 × 2.5 cm) using a thermal stimulator (MSA Thermal Stimulator, Somedic, Stockholm, Sweden). In particular, a series of 3 temperature ramps ascending from 32°C were applied to the skin. The thermode was placed on the dorsal hand skin innervated by the left superficial radial nerve or the left ulnar nerve. The forearm was laid on a table in a pronated and comfortable position. Small pillows were used when necessary. Throughout the experiment, the thermode was firmly attached to the skin by elastic straps with Velcro attachments. These were tightened just enough to hold the thermode in place without obstructing blood flow. During thermal stimulation, the rate of temperature change was 1°C/sec forward and 3°C/sec back. Skin temperature was at least 28°C. Thermal stimulation was randomized with intervals between 4 and 10 seconds. The thermal threshold was determined by using the method of limits [9, 10, 13]. The subject held a response switch in the right (dominant) hand and was asked to press it as soon as the sensation changed from one of heat to (burning) pain. This switching reversed the ascending thermal stimulation, and the turning point represented the subjective threshold expressed in degrees centigrade. An average of the three responses constituted the threshold for heat pain.

### 2.3. Electrical Stimulation

Electrical stimulation (square monophasic waveform) was generated by an electromyograph (Key-Point, Dantec-Medtronic, Skovlunde, Denmark). Two adhesive disposable surface electrodes (stimulating surface of 28 mm, 7 × 4 mm, for each electrode, Alpine Biomed ApS, Skovlunde, Denmark) were attached proximally to the left wrist (cathode placed distally), so that the superficial radial nerve was maximally stimulated along the lateral border of the radius and the paresthesia evoked was clearly felt in the nerve territory. The wrist was maintained in a neutral position. The frequency was set to 100 Hz and the pulse duration to 0.1 msec. The intensity of stimulation was increased by steps of 1 mAmp, until the sensation was considered unpleasant by the subject. Then the intensity was lowered by 1 mAmp until the paresthesia experienced was strong but not unpleasant. Electrical stimulation was never adjusted after the beginning of the experiment.

### 2.4. Protocol

A group of normal subjects underwent three different experimental sessions in a randomized order. The protocol consisted in measuring the heat pain threshold on the dorsal hand skin before, during, and after the electrical stimulation of the left radial nerve or in absence of stimulation. All subjects participated in three sessions conducted on different days. In particular, when the radial nerve was stimulated, the heat pain threshold was measured in the skin territory of the left radial nerve (session 1) or in the skin territory of the left ulnar nerve (session 2). In a third session, the heat pain threshold was measured in the skin territory of the left radial nerve without any stimulation (session 3).

In each session, the heat pain threshold was measured in basal conditions (*T*0) and after 5 (*T*1), 10 (*T*2), 15 (*T*3), 25 (*T*4), 40 (*T*5), and 70 (*T*6) minutes.

When applied, the electrical stimulation started immediately after the basal recording (*T*0) and lasted always for ten minutes.

**Figure 1:**
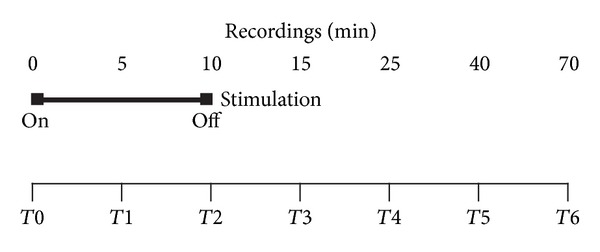
Scheme of the protocol used in the study.

A scheme of the protocol is given in Figure 1.

The protocol used in this study was approved by the Ethics Committee of the “Salvatore Maugeri” Foundation.

### 2.5. Data Analysis

Data are summarized as mean ± standard error. Trends over time of the recorded variables during the 70-minute test (7 time points from baseline to the end of test) were investigated by means of repeated measures analysis of variance with one factor (factor: group, 3 levels). 

In case of significant results (*P* value less than 0.05 for the interaction term time group), post hoc tests were performed to identify which group differs from the others. Specific contrasts to evaluate differences between subsequent time points were also investigated. Data were analysed using SPSS statistical software.

## 3. Results

According to inclusion and exclusion criteria, 10 volunteers (4 males, 6 females; mean age 36, age range 28–47) were enrolled in the study.

**Figure 2:**
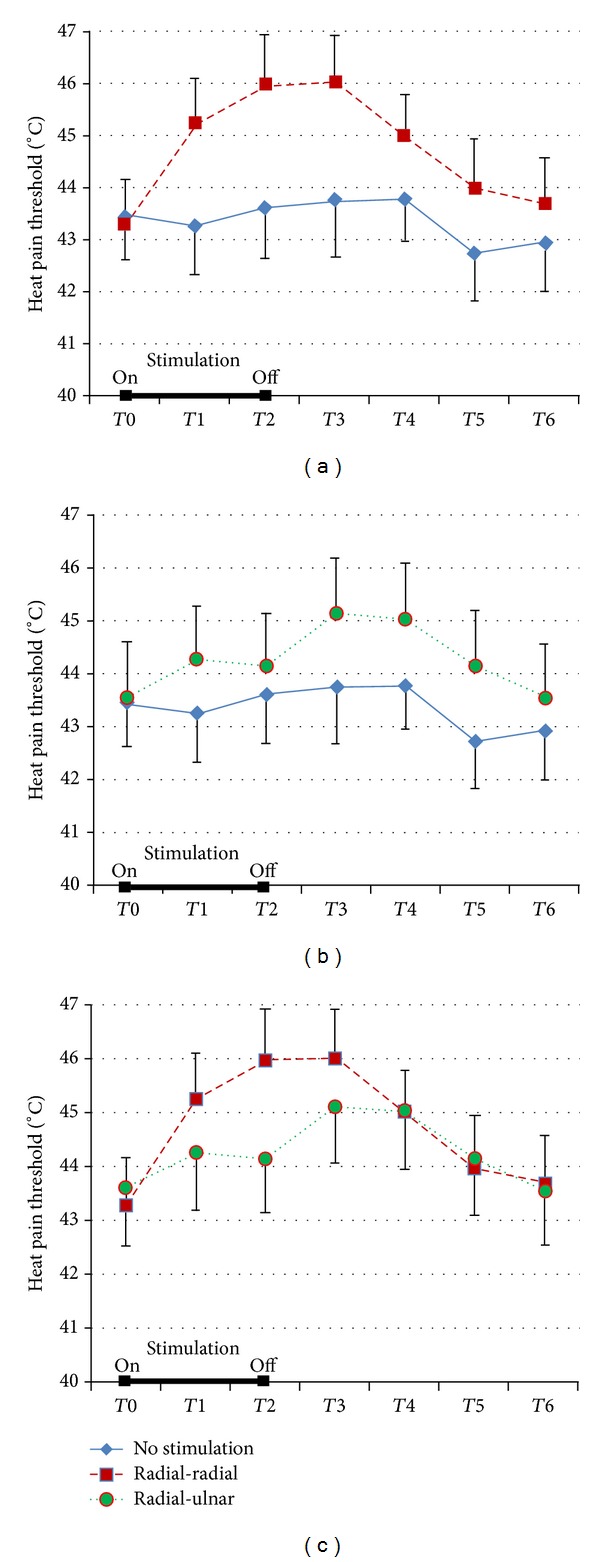
Mean and standard error of heat pain threshold before, during, and after the electrical stimulation of radial nerve. The 3 parts of the figure refer to the comparison between the 3 sessions (for further details, see the text).

Results and statistical significance are summarized in Figure 2 and Table 1.

A clear increase of heat pain threshold was observed in the skin territory of the left radial nerve when this nerve was stimulated (session 1). It started during the electrical stimulation and continued for at least five minutes after the stop. From that point, a slow return toward the basal condition was observed. Statistical analysis showed that, comparing heat pain threshold measures to the basal one, a significant increase persisted up to 15 minutes after the stimulation was stopped. 

A slight increase of heat pain threshold was also observed in the skin territory of the left ulnar nerve during and after the radial nerve stimulation (session 2), but it never reached the statistical significance when confronted to the basal value (*T*0).

No significant changes were observed in the session without stimulation (session 3).

Considering the differences between each evaluation time and the basal one, in the post hoc test (Table 1), session 1 was significantly different from session 3, except the last evaluation (*T*6 versus *T*0), suggesting a significant antalgic effect for at least 30 min after the end of the stimulation. On the contrary, the comparison between session 2 and session 3 never reached the statistical significance.

Overall, these results seem to clearly demonstrate that the antalgic effect of the electrical stimulation of the radial nerve was higher in its skin territory where it lasted for at least 30 minutes after the stimulation was stopped.

## 4. Discussion

The results obtained in the present study suggest that the antalgic effect of a nerve stimulation is higher in its skin territory than in neighbouring areas. To the best of the authors' knowledge, this is the first study that used the heat pain threshold for monitoring the antalgic effect of HF-TPNS. Previous investigations have indeed studied the changes in mechanical pain threshold [14], electrical pain threshold [15], and radiant heat pain [16]. The choice to use the heat pain threshold was justified by the classical evidence that the reduction of heat pain threshold is a hallmark of inflammation, which is the pathophysiological basis for the most part of nociceptive painful conditions that are usually treated by TENS.

The most important clinical consideration coming from the obtained results is probably the suggestion that targeting the electrical stimulation on the nerve fibres innervating the body part in pain is crucial for obtaining the most effective analgesia by TENS. Moreover, a valuable indication emerging from the higher analgesic effect obtained by the electrical stimulation of the nerve supplying the body part in pain is that the clinicians are incited to search for the nerve trunk stimulation whenever possible. In this way, it is possible not only to better target the stimulation, but also to activate a large number of nerve fibres using a reduced electrical field.

Another important result of the present study was the confirmation that the antalgic effect of HF-TPNS persists beyond the stimulation time. Similar results have indeed been previously described for the heat pain threshold [8, 17, 18] and for the pressure pain threshold [18, 19]. Different from other similar studies, the present experiment monitored the antalgic effect for one hour after the end of the stimulation, demonstrating that a significant effect persisted for at least 30 minutes after the end of the electrical stimulation and suggesting that the continuous stimulation could be unnecessary. This is a crucial clinical point because it could represent a possible practical strategy for avoiding the tolerance-like effect to repeated TENS application, recently demonstrated in humans [20]. 

Interestingly, the slight increase of heat pain threshold also observed in the skin territory of ulnar nerve during and after radial nerve stimulation suggests that the antalgic effect of HF-TPNS is at least in part a central phenomenon. A previous study came to the same conclusions using peripheral microneurographic recordings [15].

Finally, all together considered, our results also agree with an important animal study showing that the best inhibition of primate spinothalamic tract can be obtained when high-frequency, high-intensity stimulation is performed on a nerve innervating the area from which pain originates [6] and with a clinical study on peripheral neuropathic pain where the stimulation of a nerve trunk affected by painful neuropathy was significantly more effective than the stimulation of an unrelated nerve trunk [1].

## 5. Conclusions

The present study demonstrated that during and after a nerve stimulation the antalgic effect is higher in the skin territory of the stimulated nerve than in neighbouring areas. It also confirmed that the location of TENS electrodes is crucial for obtaining the strongest pain relief, advising for trunk nerve stimulation whenever possible. Moreover, the results of the present study suggest that continuous stimulation could be unnecessary, thus indicating a strategy for avoiding the well-known tolerance-like effect of prolonged TENS application. To this end, stimulation intervals lower than 30 minutes are advisable. Finally, the antalgic effect showed in the present study can be probably attributed only at high-intensity (strong but not painful), high-frequency stimulations. Further studies confronting different parameters of stimulation are warranted.

## Figures and Tables

**Table 1:** Statistical significance of differences in heat pain thresholds recorded during and after the electrical stimulation when confronted to the basal values (post hoc test included).

	Session 1, radial nerve stimulation radial territory recording	Session 2, radial nerve stimulation ulnar territory recording	Session 3, no stimulation radial territory recording
*T*1 versus *T*0	*P* = 0.001	NS	NS
*T*2 versus *T*0	*P* < 0.001	NS	NS
*T*3 versus *T*0	*P* < 0.001	NS	NS
*T*4 versus *T*0	*P* = 0.008	NS	NS
*T*5 versus *T*0	NS	NS	NS
*T*6 versus *T*0	NS	NS	NS

Post hoc test			

	Session 1 versus Session 3	Session 1 versus Session 2	Session 2 versus session 3

*T*1 versus *T*0	*P*< 0.001	NS	NS
*T*2 versus *T*0	*P* < 0.001	NS	NS
*T*3 versus *T*0	*P* < 0.001	NS	NS
*T*4 versus *T*0	*P* = 0.018	NS	NS
*T*5 versus *T*0	*P* = 0.03	NS	NS
*T*6 versus *T*0	NS	NS	NS

NS: not significant. For timing, see the text.
